# RNA-seq and flow-cytometry of conventional, scalp, and palmoplantar psoriasis reveal shared and distinct molecular pathways

**DOI:** 10.1038/s41598-018-29472-w

**Published:** 2018-07-27

**Authors:** Richard Ahn, Di Yan, Hsin-Wen Chang, Kristina Lee, Shrishti Bhattarai, Zhi-Ming Huang, Mio Nakamura, Rasnik Singh, Ladan Afifi, Keyon Taravati, Priscila Munoz-Sandoval, Mariela Pauli, Michael D. Rosenblum, Wilson Liao

**Affiliations:** 1 Department of Dermatology, University of California, San Francisco, San Francisco, CA United States; 2 School of Medicine, Case Western Reserve University, Cleveland, OH United States

## Abstract

It has long been recognized that anatomic location is an important feature for defining distinct subtypes of plaque psoriasis. However, little is known about the molecular differences between scalp, palmoplantar, and conventional plaque psoriasis. To investigate the molecular heterogeneity of these psoriasis subtypes, we performed RNA-seq and flow cytometry on skin samples from individuals with scalp, palmoplantar, and conventional plaque psoriasis, along with samples from healthy control patients. We performed differential expression analysis and network analysis using weighted gene coexpression network analysis (WGCNA). Our analysis revealed a core set of 763 differentially expressed genes common to all sub-types of psoriasis. In contrast, we identified 605, 632, and 262 genes uniquely differentially expressed in conventional, scalp, and palmoplantar psoriasis, respectively. WGCNA and pathway analysis revealed biological processes for the core genes as well as subtype-specific genes. Flow cytometry analysis revealed a shared increase in the percentage of CD4+ T regulatory cells in all psoriasis subtypes relative to controls, whereas distinct psoriasis subtypes displayed differences in IL-17A, IFN-gamma, and IL-22 production. This work reveals the molecular heterogeneity of plaque psoriasis and identifies subtype-specific signaling pathways that will aid in the development of therapy that is appropriate for each subtype of plaque psoriasis.

## Introduction

While the skin lesions of plaque psoriasis are described classically as well-circumscribed, raised, erythematous lesions with overlying silvery scale, there is considerable heterogeneity in the clinical presentation of the disease. The anatomic location of psoriatic lesions, time course (chronic vs. acute), age of onset (adult vs. pediatric), lesion morphology (plaque size, induration, erythema, and scale), and response to therapy may vary from patient to patient. This varied spectrum of clinical phenotypes suggests that psoriasis may comprise a collection of several different biologic subtypes, which while sharing certain common features, may also have unique immunogenetic characteristics. While “conventional” plaque psoriasis typically affects the trunk and extensor surfaces (elbows/knees), other patterns of lesion distributions such as scalp and palmoplantar psoriasis are recognized as clinically distinct subtypes of plaque psoriasis^1^. Scalp and palmoplantar psoriasis may each occur in isolation or with other psoriasis subtypes, but tend to be more refractory to treatment compared to conventional plaque psoriasis^2,3^. These two subtypes are also associated with a higher burden of disease that is both greater than conventional psoriasis and disproportionate to the body surface area affected^4^. For instance, patients with scalp and palmoplantar psoriasis report greater physical and psychosocial impairment from their disease compared to patients with the conventional plaque form of psoriasis^4–6^. Although physicians recognize the clinical differences between these subtypes, little is known about the molecular and transcriptomic differences between conventional, scalp, and palmoplantar psoriasis, especially with respect to key populations of immune cells. In this study, we use RNA-seq to assess the transcriptomic differences between scalp, palmoplantar, and conventional psoriasis and use flow cytometry to profile the cytokines secreted by CD4+ T effector cells, CD8+ T effector cells, and CD4+ regulatory T cells (Tregs).

## Methods

### Study population

We recruited 8 patients with conventional plaque psoriasis (CP) on the trunk and extremities, 8 patients with scalp psoriasis (SP), and 3 patients with plaque-type palmoplantar/handfoot psoriasis (HF) from the San Francisco Bay area. Subjects were adults over the age of 18 who had a diagnosis of psoriasis from a board certified dermatologist for at least 6 months (mean duration = 19.9 years) and who were not on systemic treatments. For subjects on topical therapy, the sampled plaque was free of topical therapy for at least 2 weeks prior to the biopsy procedure. Normal healthy skin from the surgical discards of 9 cosmetic surgery patients was used as controls (C). Skin from surgical discards came from the trunk or upper legs. This study was approved by the University of California, San Francisco’s institutional review board and all subjects provided written informed consent prior to enrollment. All subjects were de-identified and any HIPAA identifiers were removed.

### Skin biopsies

Two 4-mm punch biopsies were obtained from the lesional skin of each psoriasis patient, with one biopsy used for RNA-seq and the other for flow cytometry. Biopsies of conventional plaque psoriasis were taken from lesions on the arms or legs. Tissue samples were placed either into RNALater solution or onto sterile gauze that had been dampened with phosphate buffered saline and stored at 4 °C until further processing by flow cytometry.

### RNA sequencing

RNA was extracted from one 4-mm punch biopsy specimen for bulk RNA sequencing with the Qiagen Allprep DNA/RNA mini kit. The Agilent 2100 bioanalyzer was used to perform quality control and identify samples with a high proportion of minimally degraded mRNA (defined as an RNA integrity number >7.500 pg). Samples that passed quality control underwent library preparation using the Ovation RNA-Seq System V2 protocol (Nugen) or the Ribozero (Illumina) kits. Paired-end sequencing with read length of 100 bp was conducted using the Illumina Hiseq platform.

#### Read alignment, quality control, and differential expression analysis

**Figure 1:**
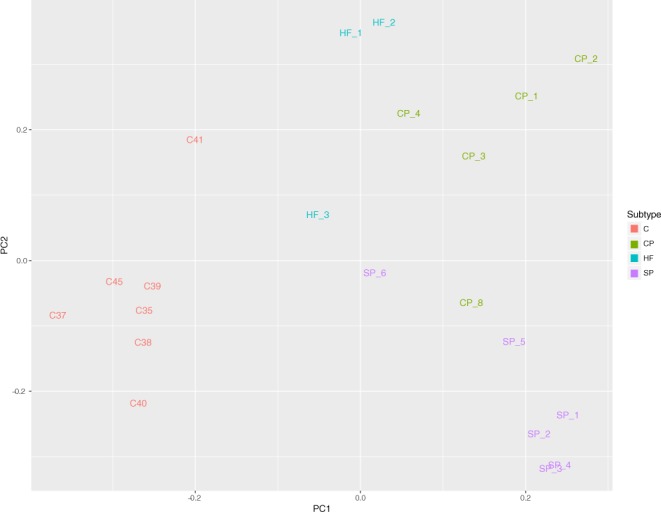
PCA plot of all samples, after detection and removal of outliers.

Read quality was checked using FastQC v. 0.11.4. Reads were aligned to the UCSC hg19 human reference genome using STAR v. 2.4.2^7^. Principal components analysis was performed to detect outliers and any batch effect (Fig. 1). For the RNA-seq analysis only, we removed 2 healthy controls, 3 CP, and 2 SP samples due to sequencing batch effect. The ComBat function in the sva package^8^ was implemented to adjust for residual sequencing batch effect of the remaining samples. The Cufflinks package v. 2.2.1^9^ was implemented to perform normalization and test for differential expression. BioVenn^10^ was implemented to create an intersectional plot of differentially expressed genes in each psoriasis subtype.

#### Network analysis

We performed further quality-control on the matrix of normalized expression values to remove any transcripts with either zero variance or a missing value prior to performing weighted gene coexpression network analysis (WGCNA)^11^. We implemented the WGCNA package to create a weighted adjacency matrix. The soft thresholding parameter, β, for the power function was set to 12 after a sensitivity analysis of scale-free topology was performed (Supplementary Fig. 1). This weighted adjacency matrix was used to generate a topological overlap matrix (TOM) and dendrogram. A dynamic hybrid branch cutting method was implemented on the resulting TOM-based dendrogram to identify modules. A cut height of 0.3 was set to merge module eigengenes (ME; first principal component of each network) that have a correlation of 0.7 or greater. We then estimated MEs, which are the first principal components for each gene expression module after a singular value decomposition is performed on the TOM. We calculated gene significance, defined as *GS*_*i*_ = |cor*(*x*_*i*_*, t*)|, where *x*_*i*_ is the *i-*th gene and *t* is the binary indicator variable for phenotype. We also calculated the ME significance, defined as *MES*_*i*_ = |correlation (*ME*_*j,t*_)|, where *ME*_*i*_ is the *j*-th ME. Module membership, MM, for the ith gene was defined as, MM = |correlation (*x*_*i*_, *ME*)|.

### Flow cytometry

One 4 mm skin punch biopsy was taken from each patient and immediately stored at 4 °C in a container with sterile gauze and PBS until it was ready to be processed. The tissue was trimmed to remove hair and subcutaneous adipose before being finely minced and mixed with digestion buffer composed of Collagenase Type IV (Worthington LS04188), DNAse (Sigma-Aldrich DN25-1G), 10% FBS, 1% HEPES, and 1% Penicillin/Streptavidin in RPMI 1640. After overnight incubation in 5% CO_2_, cell suspensions were harvested, filtered, centrifuged, and counted. Cells were then stimulated and incubated for 4 hours with Cell Stimulation Cocktail 500× (Tonbo biosciences; TNB-4975). Cells were stained with viability dye Ghost Dye^TM^ Violet 510 (Tonbo biosciences 130870) and the following antibodies: anti-hCD3 PerCP (Biolegend; SK7), anti-hIL-13 FITC (eBioscience; 85BRD), anti-hTNF-α PE-Cy7 (BD Pharmigen; MAb11), anti-hCD4 PE-Texas Red (Invitrogen; S3.5), anti-hIL-22 PE (R&D Systems; 142928), anti-hCD45 APC-eFluor 780 (eBioscience; 2D1), anti-hIFN-γ Alexa Fluor 700 (Biolegend; 4S.B3), anti-hIL-17A eFluor 660 (eBioscience; eBio64CAP17), anti-Foxp3 eFluor 450 (eBioscience; PCH101), and anti-hCD8a eVolve 605 (eBioscience; RPA-T8). We then performed multi-parameter flow cytometry to subset the leukocyte population into defined T cell populations (CD4+ T effectors, CD8+ T cells, and CD4+ Foxp3+ Tregs) based on cell surface and intracellular differentiation/activation markers and quantified the cytokines produced by each cell type. Data was acquired using the LSR Fortessa (BD Biosciences) flow cytometer and results were analyzed using FlowJo software platform (Tree Star Inc.) (Supplementary Fig. 2).

### Statistical analysis

Cytokine and chemokine levels were compared with the Mann-Whitney U test in GraphPad Prism 7.

### Data Availability Statement

RNA-seq data generated from this study has been deposited in Gene Expression Omnibus and are accessible through GEO Series accession number GSE117405 (https://www.ncbi.nlm.nih.gov/geo/query/acc.cgi?acc=GSE117405).

## Results

### Patient demographics

A summary of patient demographic characteristics is provided in Table 1. Age, psoriasis age of onset, and body mass index (BMI) were comparable among subjects with conventional psoriasis, scalp psoriasis, and healthy controls. For gender, the control group had a higher proportion of female subjects compared to the conventional plaque group. The palmoplantar psoriasis group was not included in the statistical analyses of demographic features due to small sample size.

**Table 1:** Summary of patient demographic characteristics.

	Conventional Psoriasis (CP)	Scalp psoriasis (SP)	Palmoplantar psoriasis (HF)	Controls (C)	P-value
Age (Mean, Range)	51.5 (35–74)	59 (32–72)	49 (33–65)	42 (20–73)	CP vs SP: p = 0.528
					CP vs C: p = 0.311
					SP vs C: p = 0.122
Gender (F:M)	1:7	4:4	2:0	7:2	CP vs SP: p = 0.282
					CP vs C: p = 0.015*
					SP vs. C: p = 0.335
Age of onset (Mean, Range)	30.5 (1–62)	38.5 (3–58)	38.5 (28–49)	N/A	CP vs SP: p = 0.563
Duration of disease (Mean, Range)	25.2 (8–60)	18.1 (3–56)	10.5 (5–16)	N/A	CP vs SP: p = 0.316
BMI (Mean, Range)	26.7 (24.3–37.2)	25.7 (18.6–27.6)	31.3 (22.7–39.9)	N/A	CP vs SP: P = 0.195

Unadjusted p-values are derived from pair-wise comparisons of psoriasis subtypes by the Mann-Whitney U test for continuous variables and Fisher’s exact test for categorical variables. The palmoplantar psoriasis group was not included in the statistical analyses due to small sample size. *p ≤ 0.05.

### RNA sequencing

#### Differential expression analysis reveals core set of shared genes involved in conventional, scalp, and palmoplantar psoriasis

**Figure 2:**
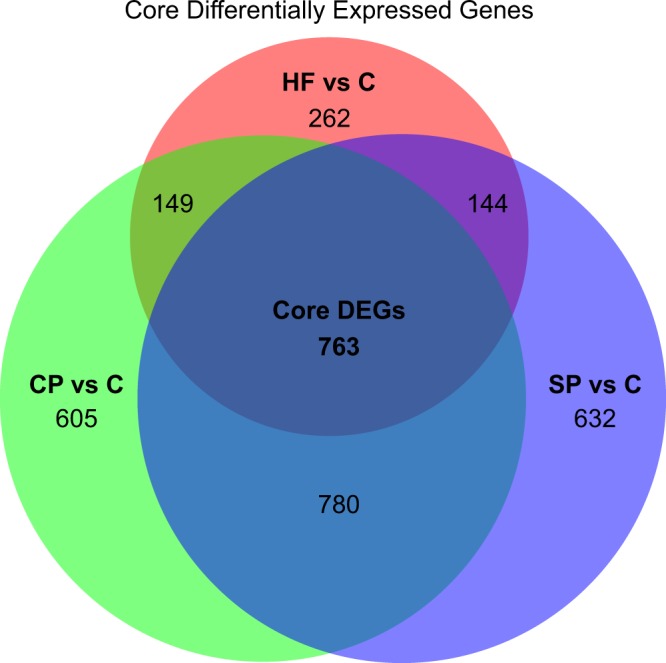
Venn diagram showing the number of core DEGs, which are the intersection of DEGs between HF vs C, CP vs C, and SP vs C. Circles are sized according to the number of DEGs in each test and the size of the intersections are sized according to the number of intersections between each test.

**Figure 3:**
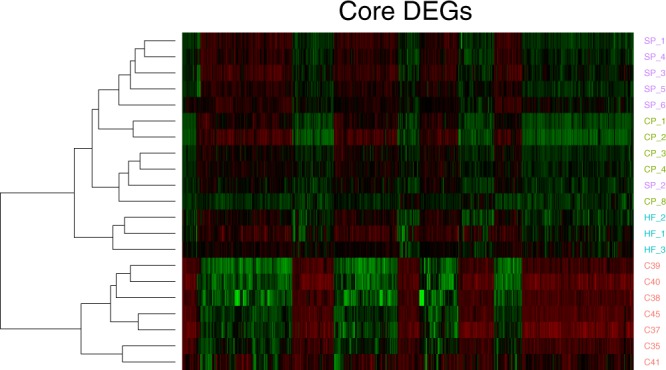
A heatmap of the gene expression of the core 763 DEGs, showing clear separation between all subtypes of psoriasis and healthy controls and clustering of subtypes of psoriasis.

**Figure 4:**
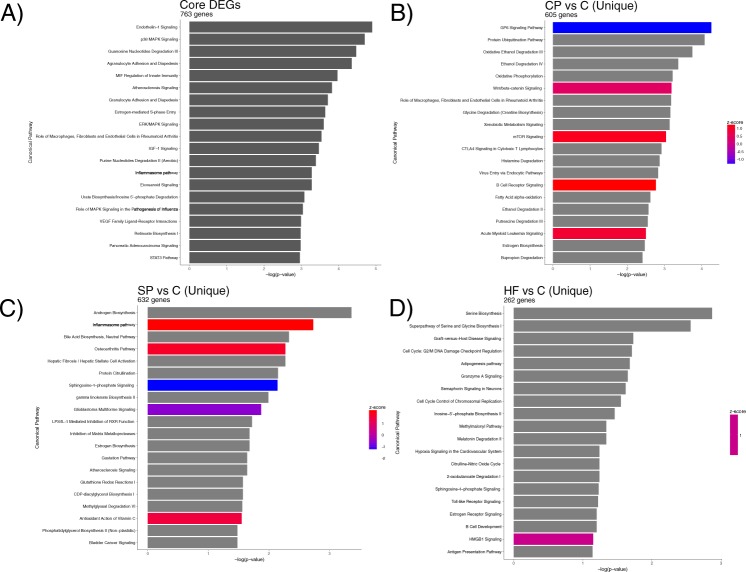
Barplots of Ingenuity Pathway Analysis canonical pathways enriched for in the Core DEGs (**A**), CP vs C (**B**), SP vs C (**C**), HF vs C (**D**).

Differential expression analysis revealed 2297 differentially expressed (DE) genes (DEGs) in CP vs C, 2319 DEGs in SP vs C, and 1318 DEGs in HF vs C (|log_2_(FC)| ≥ 1 and q ≤ 0.05; Table 2, Supplementary Table 1). An intersection of all DEGs from CP vs C, SP vs C, and HF vs C revealed a core set of 763 DEGs common to each subtype of psoriasis (Fig. 2, Supplementary Table 2). Top DEGs from this core set include genes such as *S100A7A*, *SPRR2A/B*, *SERPINB4*, *S100A9*, *KRT6*, *C10orf99*, *LCE3D/E*, *IL36G*, and *OASL*, which have been found to be DE in previous studies of CP^12,13^, SP^14^, and HF^15^. A heatmap of this core set of DEGs reveals a clear distinction between psoriasis and healthy control samples (Fig. 3). Interestingly, this core set of genes also results in differential clustering of psoriasis subtypes, suggesting that these core genes may have different expression levels among psoriasis subtypes. Top Ingenuity Pathway Analysis (IPA; www.qiagen.com) canonical pathways enriched for with the core DEGs include “Endothelin-1 Signaling”, “p38 MAPK Signaling”, “MIF Regulation of Innate Immunity”, and “Atherosclerosis Signaling” (−log (p-value) ≥ 1.3; Fig. 4A, Supplementary Table 3).

**Table 2. Number of differentially expressed genes (DEGs) in CP vs C, SP vs C, and HF vs C.** *|log_2_(FC)| ≥ 1 and q ≤ 0.05.

Comparison (A vs B)	Number DEGs*	Up-regulated in A	Down-regulated in A
CP vs C	2319	1119	1200
SP vs C	2297	1128	1169
HF vs C	1318	774	544

#### Conventional psoriasis transcriptome

Top up-regulated DEGs in CP vs C include previously identified psoriasis-associated genes such as *IL36A/G*, *SPRR2A/B/F*, *SERPINB4*, *S100A7A*, *S100A9*, and *IL17F* while top down-regulated DEGs include *SERTM1*, *IL6*, and *ADAMTS16* (Supplementary Table 1). Amongst the 2297 DEGs in CP vs C, 605 DEGs were exclusive to CP vs C (Supplementary Table 4). Amongst these CP vs C exclusive genes, top up-regulated genes include *SST*, *TTTY14*, *PRKY*, *IL20*, *KRT33A*, and *HOXD11* while top down-regulated genes include *SERTM1*, *ADAMTS16*, *MATN4*, and *HAS1*. IPA canonical pathways enriched for amongst these CP vs C exclusive DEGs include “GP6 Signaling Pathway”, “Wnt/Β-catenin Signaling”, “mTOR Signaling”, and “B Cell Receptor Signaling” (−log (p-value ≥ 1.3; Fig. 4B, Supplementary Table 3).

#### Scalp psoriasis transcriptome

In SP vs C, a majority (1543/2319 or 67%) of the DEGs were also differentially expressed in CP vs C, including known CP signature genes such as *S100A7A*, *SPRR2B*, *IL36A/G*, *IL17D/F/RD/RE* and *SERPINB4*. Keratin (*KRT*-) and keratin-associated protein (*KRTAP*-) family genes were also highly expressed in SP and in many cases, DE between SP and C, much more so than in CP or HF. While these *KRT-* and *KRTAP-* genes are likely to be highly important in the normal development of hair follicles and hair, results from a previous study of SP by Ruano *et al*.^14^ that compared lesional scalp tissue to normal scalp tissue did not find evidence that keratin or keratin-associated protein genes are DE. Nevertheless, 197/1128 of the up-regulated genes in SP were also up-regulated in SP skin in Ruano *et al*.^14^. 632 of the DEGs were exclusive to SP vs C and included top up-regulated genes *LCE3C*, *FAM26D*, and *CBLN2* while top down-regulated genes included *AGR3*, *TBX5*, and *HOXA10* (Supplementary Table 4). Top IPA canonical pathways enriched for in DEGs unique to SP include “Androgen Biosynthesis”, “Inflammasome Pathway”, and “Osteoarthritis Pathway” (−log (p-value) ≥ 1.3; Fig. 4B, Supplementary Table 3).

#### Palmoplantar psoriasis transcriptome

Relative to SP and CP, HF was transcriptomically closest to C and nearly 60% of the 1318 DEGs in HF vs C are members of the core set of DEGs common to all subtypes of psoriasis. In contrast, only about 33% of the DEGs in CP vs C and SP vs C are members of the psoriasis core DEG set. Of the 1318 DEGs, 262 were unique to HF vs C with top up-regulated genes including *S100A7*, *NEFL*, and *SERPINB3* while top down-regulated genes included *KRT25*, *TCHH*, and *CDH22* (Supplementary Table 4). Top IPA canonical pathways enriched for in DEGs unique to HF revealed that the top enriched pathways include “Serine Biosynthesis”, “Graft-versus-Host Disease Signaling”, and “Semaphorin Signaling in Neurons” (−log (p-value) ≥ 1.3; Fig. 4C, Supplementary Table 3).

#### Weighted gene co-expression network analysis (WGCNA) reveals SP and HF specific networks of co-expressed genes

**Figure 5:**
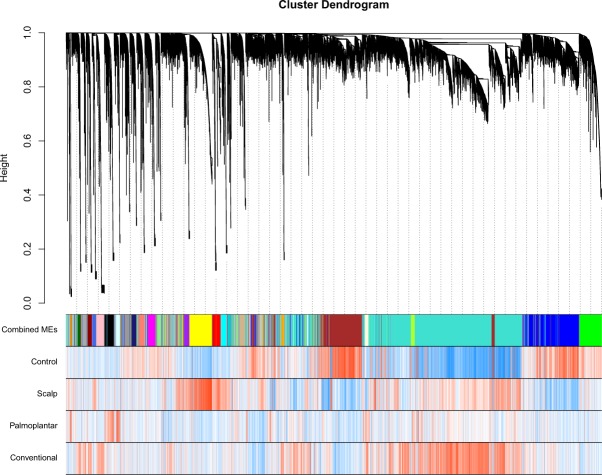
Hierarchical clustering dendrogram of modules identified by WGCNA with branches corresponding to module color assignments in the first color band beneath the dendrogram. The remaining color bands show how positively (red) or negatively (blue) an individual gene is correlated with a specific module and cell type.

To identify genes that are co-expressed within networks of genes correlated with each subtype of psoriasis, we performed a weighted gene co-expression network analysis (WGCNA). After construction of the co-expression network and subsequent branch pruning, we were left with 28 networks (Fig. 5). As WGCNA is an unsupervised learning method, to determine which networks are associated with each specific sub-type of psoriasis, we calculated the module eigengene (ME), or the first principal component of each network, and performed a Pearson correlation between each ME and CP, SP, and HF. We found 2/28 modules significantly correlated modules (Pearson ρ >  = 0.7 and q < 0.05), one correlated with SP (q = 8.31 × 10^−7^) and one with HF (q = 6.05 × 10^−6^).

**Figure 6:**
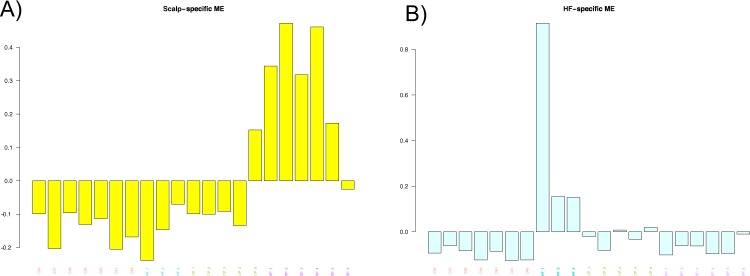
Barplots of ME expression across all samples for modules that are associated with SP (**A**) and HF (**B**).

As the ME is the first principal component of a module, it can be seen as a proxy for the overall expression profile of a given module. The two modules that are correlated with SP and HF, respectively, are the yellow (n_genes_ = 1200) and lightcyan (n_genes_ = 260) modules (see Supplementary Table 4 for all genes in each module). Barplots visually capture the distinct patterns of expression in the SP and HF modules (Fig. 6A,B). The canonical pathways that the SP module hub genes were significantly enriched for were “Glucocorticoid Receptor Signaling”, “Protein Citrullination”, and “1,25 – Dihydroxyvitamin D3 Biosynthesis” while the canonical pathways that the HF module hub genes were significantly enriched for were “Pregnenolone Biosynthesis”, “Histidine Degradation VI”, and “Ubiquinol-10 Biosynthesis” (−log (p-value) ≥ 1.3).

To identify the most influential genes within each module, or the “hub” gene, we calculated the module membership (MM) for each gene. We used a threshold of MM ≥ 0.8 to identify hub genes and identified 58 hub genes in the HF module and 361 hub genes in the SP module (see Supplementary Table 5 for all hub genes). Some of the most influential genes within the HF module, such as *NEFL* and *ALOX15* are DEGs in HF vs C. However, a majority of the hub genes were not DE in any of the comparisons.

### Flow Cytometry Profiles

#### Psoriatic skin vs healthy skin

**Figure 7:**
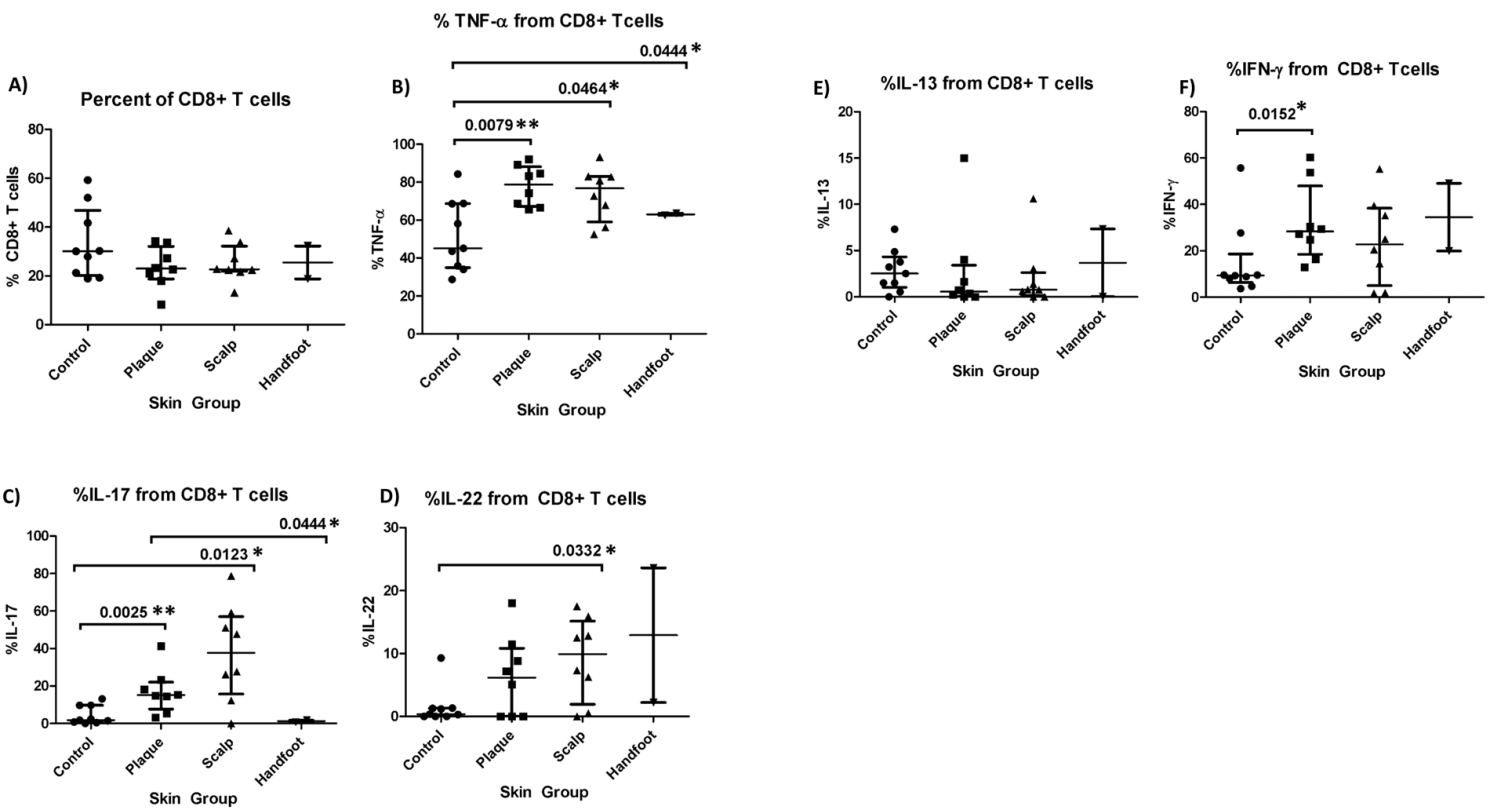
Percentage of CD8+ T cells and CD8+ T-cell derived cytokines in healthy skin and lesional skin from conventional plaque, scalp, and hand foot subtypes of psoriasis. Bars represent the median and interquartile range.

**Figure 8:**
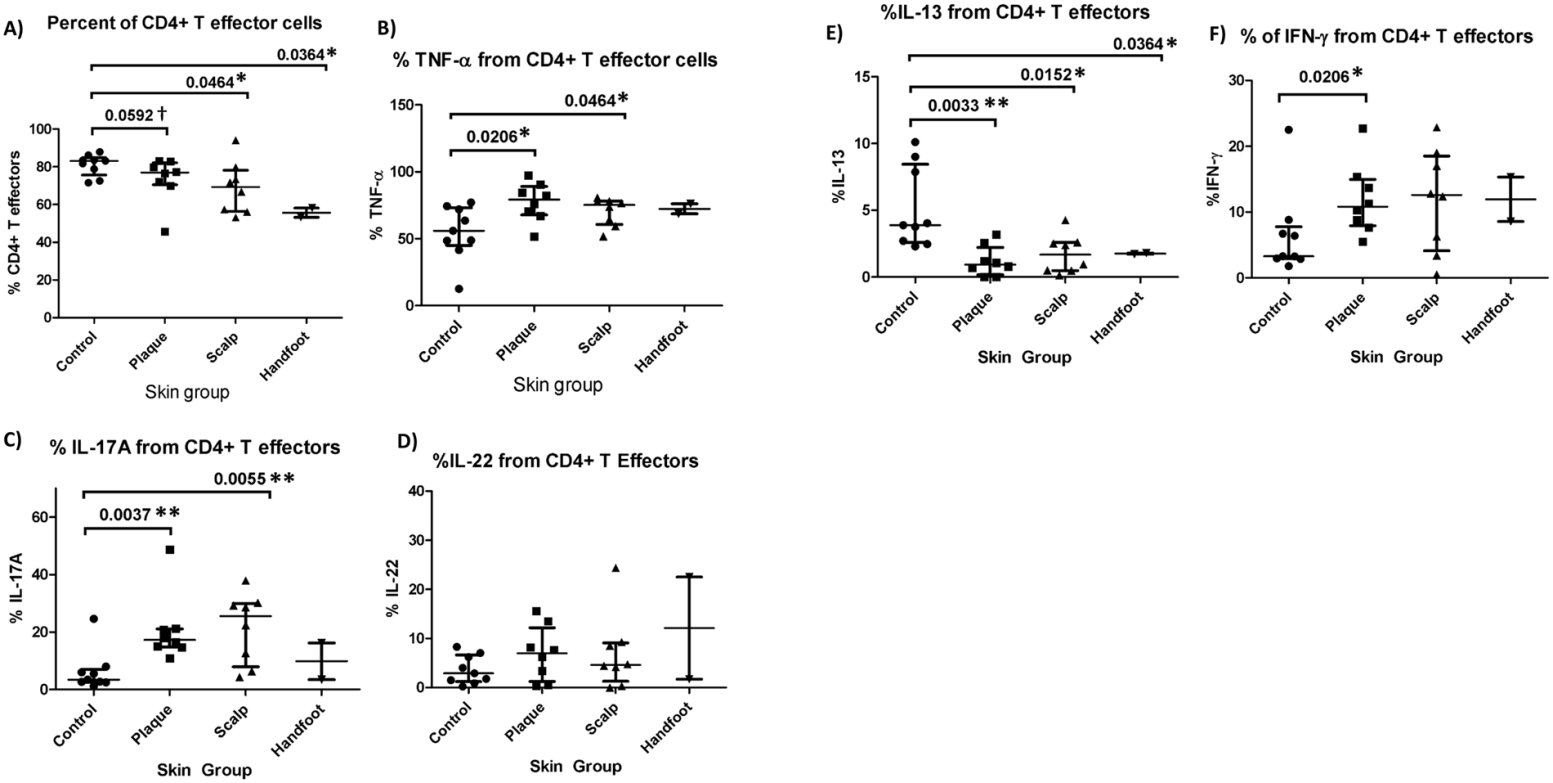
Percentage of CD4+ T effector cells and CD4+ T-effector cell derived cytokines in healthy skin and lesional skin from conventional plaque, scalp, and hand foot subtypes of psoriasis. Bars represent the median and interquartile range.

**Figure 9:**
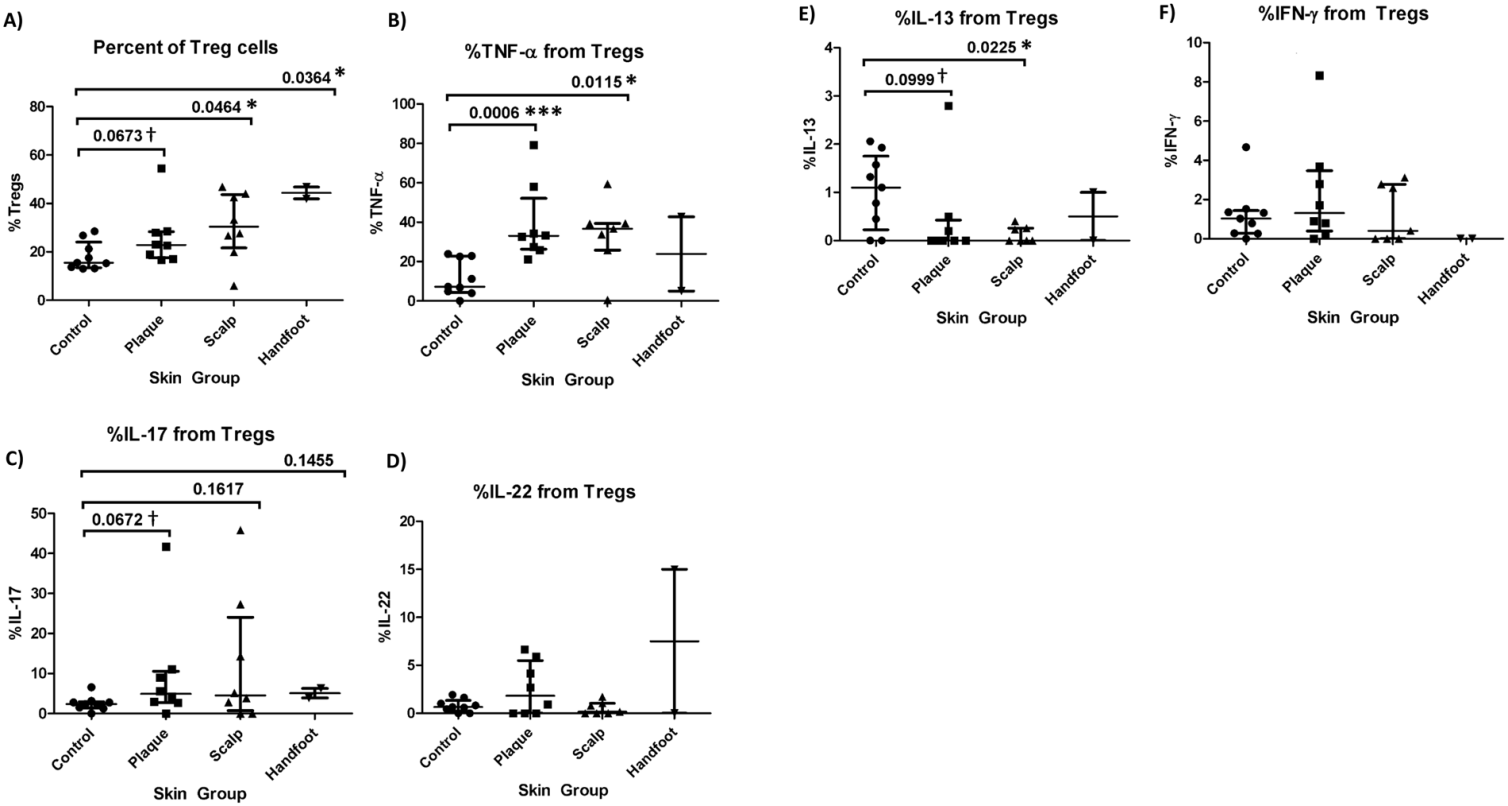
Percentage of CD4+ T regulatory cells and CD4+ T regulatory cell derived cytokines in healthy skin and lesional skin from conventional plaque, scalp, and hand foot subtypes of psoriasis. Bars represent the median and interquartile range.

We first examined whether there were commonalities between all three subtypes of psoriasis and healthy skin. In terms of T cell composition, the percentage of CD4+ Tregs out of total T cells was consistently higher in each of the three psoriasis subtypes compared to control skin, whereas the percentage of CD4+ T effectors was lower than control skin, and percentage of CD8+ T effectors the same as control skin (Figs 7A and 8A). Furthermore, the percentage of TNF-α derived from CD8+ T cells, CD4+ T effectors, and CD4+ Tregs, were significantly higher in conventional plaque and scalp psoriasis than in control skin (Figs 7B, 8B and 9B). CP and SP also had a higher percentage of IL-17A from CD8+ T cells and CD4+ T effectors than control skin (Figs 7C and 8C). There was a trend towards an increased percentage of IL-17A from CD4+ Treg cells in psoriatic skin (Fig. 9C). All three psoriasis subtypes had significantly lower IL-13 from CD4+ T effector cells compared to healthy control skin (Fig. 8E). The higher percentage of IL-13 from psoriatic CD4+ Tregs was significant or nearly significant compared to control Tregs (Fig. 9E).

#### Differences between psoriasis subtypes

**Figure 10:**
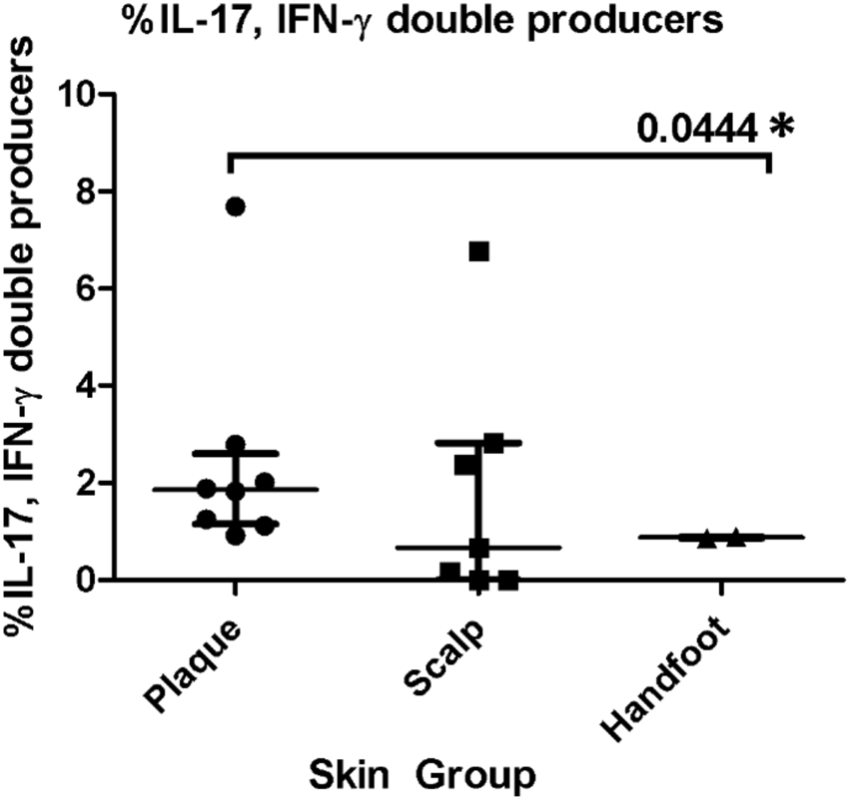
Percentage of IL-17, IFN-γ double producers in the lesional skin of the conventional plaque, scalp, and handfoot subtypes of psoriasis.

Conventional plaque psoriasis had a significantly higher percentage of IL-17A and TNF-α from CD8+ T cells relative to palmoplantar psoriasis (p = 0.044 and p = 0.044 respectively) (Fig. 7B,C). Plaque psoriasis also had more IL-17A and IFN-γ double producers than palmoplantar psoriasis (p = 0.044) (Fig. 10). Conventional plaque psoriasis had a significantly higher percentage of IFN-γ from CD4+ T cells and CD8+ T cells compared to controls (p = 0.021 and p = 0.015, respectively), while patients with scalp psoriasis did not differ appreciably from controls (Figs 7F and 8F). In contrast, only scalp psoriasis had a higher percentage of IL-22 from CD8+ T cells relative to controls (p = 0.029) (Fig. 7D).

#### Differential expression between high and low IL-17A producing CD8+ T cells

Differential expression analysis between samples with a high and low proportion of IL-17A producing CD8+ T cells (IL17A_Hi_ and IL17A_Lo_, respectively–see Fig. 7C) revealed 83 genes differentially expressed (|log_2_(FC)| ≥ 1 and q ≤ 0.05) between IL17A_Hi_ and IL17A_Lo_ samples, including 25 up-regulated in the IL17A_Hi_ group (see Supplementary Table 6 for a full list of differentially expressed genes; Supplementary Fig. 3). Top up-regulated genes include *SFRP4*, which was interestingly found to be down-regulated in psoriasis skin^16^, and *CCL13*, which was also found to be down-regulated in lesional psoriasis skin^17^.

## Discussion

### RNA-seq analysis

In our analysis, we identified a core set of psoriasis genes that are common to CP, SP, and HF. These genes include genes that are well known to be involved in psoriasis, including *LCE3D*, *LCE3E*, *SERPINB4*, and *S100A7A*, genes involved in activation and proliferation of keratinocytes, modulation of host immune response, and antimicrobial defense. Notably, two members of the IL-1 superfamily of cytokines are represented, including *IL36B* and *IL36G*. IL-34, a cytokine that has recently been found to be elevated in the serum of psoriasis patients (particularly in those with psoriatic arthritis)^18^, was the only other interleukin cytokine that was in the core set. Recently, much attention has been focused on the role of IL-36 genes in psoriasis pathogenesis, especially as a biomarker^19^ and as a potential therapeutic target^20^. IL-36 cytokines have been shown to trigger the activation of the NF-kB pathway as well as the mitogen-activated protein kinase (MAPK) pathway, one of the top canonical pathways enriched for with the core DEGs. Most recently, Arakawa *et al*.^21^ demonstrated that IL-36 signaling facilitates the Th17 responses in generalized pustular psoriasis. *IL36B* and *IL36G* are both in the core set of DEGs and highly up-regulated in each form of psoriasis. Interestingly, *IL36RN*, the gene encoding the IL-36 receptor antagonist, is also highly up-regulated in all forms of psoriasis.

One of the top IPA canonical pathways enriched for with the core set of DEGs is endothelin-1 signaling. Endothelin-1 is a peptide produced by endothelial cells with systemic vasoconstrictor properties that is associated with mitogenic action in smooth muscle cells as well as endothelial cells. Within the skin, endothelin-1 is present and functions within the microvasculature. Cecchi *et al*.^22^ observed that patients with psoriasis had much higher serum plasma levels of endothelin-1 and more recently, Borska *et al*.^23^ also observed higher serum plasma levels of endothelin-1 in psoriasis patients versus healthy control patients. Nakahara *et al*.^24^ recently discovered that endothelin-1 polarizes the dendritic cell – T cell response towards Th17/Th1 differentiation and induces dendritic cell production of pro-inflammatory cytokines such as IL-12, IL-23, and IL-6. They also showed via immunohistochemical that endothelin-1 is expressed primarily in basal keratinocytes and more highly expressed in severe cases of psoriasis.

Regarding scalp psoriasis, our results are similar to those of Ruano *et al*.^14^, who concluded that the SP transcriptome is similar to the CP transcriptome but with more prominent expression of key psoriatic genes in CP. We found that not only was the direction of expression of the overlapping DEGs between CP vs C and SP vs C the same, but also that the mean FPKM value for these overlapping DEGs was slightly higher in CP (mean = 48.7) than in SP (mean = 45.0). One of the key genes in the top unique SP vs C pathways was *AKR1C3*. Aldo-keto reductase 1C3 (*AKR1C3*) is a gene that is expressed in the epidermis that mediates the metabolism of steroid hormones and has been shown to affect keratinocyte differentiation and is up-regulated in atopic dermatitis^25^ but not in psoriasis. Interestingly, *AKR1C3* is significantly down-regulated in SP vs C.

In palmoplantar psoriasis, a number of the up-regulated genes in HF in our study were found to be up-regulated in normal palmoplantar skin (relative to normal non-palmoplantar skin) in a previous study by Bissonette *et al*.^15^, including *S100A7/8/9*, *DEFB4A/B*, *PI3*, *IL36G*, *SERPINB4*, and *KRT9*. While many of these up-regulated genes in normal palmoplantar skin (relative to normal non-palmoplantar skin) were also found to be up-regulated in HF skin by Bissonette *et al*.^15^, 16 of these up-regulated genes in normal palmoplantar skin—including genes up-regulated in CP such as *KRT9*, *KRT16*, *S100A7*, *S100A8*, and *WNT5A*—were not DE in their comparison between HF and normal palmoplantar skin. Two of the most up-regulated genes in Bissonette *et al*.^15^, GPRIN1 and ADAM23, were also up-regulated in HF vs C. However, neither of these genes were amongst the top-most up-regulated genes in HF in our analysis. Semaphorin-4D (*SEMA4D*; also known as *CD100*) is a key gene in the top HF vs C pathway, “Semaphorin Signaling in Neurons”, was significantly up-regulated in HF vs C. Interestingly, Zhang *et al*.^26^ recently showed that *SEMA4D* promotes inflammation by activating the NF-kB and inflammasome pathways in keratinocytes. *IL33* is a key gene in the HF vs C pathway, “Graft-versus-Host Disease Signaling” that was amongst the newly identified interleukin cytokines involved in psoriasis by Li *et al*.^18^ that was found to be elevated in the serum of psoriasis and psoriatic arthritis patients. However, we found that *IL33* is significantly down-regulated in HF.

### T cell production of TNF-α and IL-17A

Based on our flow cytometry data, TNF-α appears to have a central role in the pathogenesis of all plaque subtypes. Previous studies have demonstrated elevated levels of TNF-α in the serum^27,28^ and skin of psoriasis patients^29^. Moreover, anti-TNF-α biologics are an effective treatment for psoriatic disease. Our study shows that CD8+ T cells, CD4+ T effectors, and CD4+ Tregs all contribute to an increase in TNF-α in psoriatic skin, suggesting multiple elements of immune dysfunction. While keratinocytes do not respond only to TNF-α, high levels of TNF-α in conjunction with elevated IL-17A stimulate keratinocyte hyperproliferation and drive the inflammatory feedback loop in psoriasis^30^. In psoriasis, TNF-α is thought to work synergistically with IL-17A by inducing IL-17 receptor (IL-17R) expression on keratinocytes, promoting the maturation of Th17 and Th22 cells, and increasing the production of IL-17A^31^. In fact, our study shows that IL-17A from CD8+ T cells, CD4+ T effectors, and CD4+ Tregs were also higher in psoriasis samples compared to controls. The increase in TNF-α and IL-17A from CD4+ T effectors is accompanied by an increase in the proportion of CD4+ T effectors in psoriatic skin. These findings likely reflect an expansion of the Th17 subset of CD4+ T effectors, which act as “professional” IL-17A producers that, in psoriasis, create a positive feedback loop with other pro-inflammatory cytokines such as IL-23^32^.

However, the increased percentage of CD4+ Treg-derived TNF-α and IL-17A is more surprising. Normally, CD4+ Tregs modulate inflammation and promote tolerance to self-antigens by suppressing the activation and proliferation of CD4+ and CD8+ T cells. However, in a pro-inflammatory environment, Tregs may be converted to a phenotype that co-expresses FOXP3 and RORγt, a transcription factor typically associated with the Th17 lineage^33^. These FOXP3+RORγt+ Treg cells have been shown to produce IL-17 and propagate the inflammatory milieu^34^. While in our study the trend towards increased Treg-derived IL-17A in psoriasis was not statistically significant (p = 0.063 conventional plaque, p = 0.16 scalp, p = 0.15 palmoplantar), the relatively small number of patients in our study limited our power. In addition to IL-17A, our study suggests that some pathogenic Tregs may also produce more TNF-α in psoriasis skin, potentiating the effects of IL-17A and contributing further to the inflammatory cascade. Furthermore, there is also an increased percentage of CD4+ Treg cells in psoriatic skin. This is consistent with prior studies, which also found that disease severity was inversely correlated with the ratio of Th17 to Treg cells in the skin^35^. The expansion of Tregs in psoriatic skin suggests an impairment of their typical immunosuppressive functions^33^. Indeed, studies have shown that in psoriasis, Treg suppression of pathologic effector CD4+ T cells is impaired^33,36,37^. Tregs may, therefore, be a novel, unexplored therapeutic target for the treatment of plaque psoriasis.

Increases in CD8+ T cell derived TNF-α and IL-17A also intriguing as psoriasis has traditionally been thought of as a Th1/Th17 mediated disease. Elevated numbers of TNF-α and IL-17A producing CD8+ T cells have only recently been found in the skin^38,39^ and synovial fluid of patients with psoriatic disease^35^ and their role in the disease process has not been fully elucidated. Interestingly, this population of CD8+ T cells is resistant to immunomodulation by Tregs^38^. Thus, the production of TNF-α and IL-17A by CD8+ T cells can go unchecked in psoriatic skin, despite a normal percentage of CD8+ T cells relative to controls. In fact, treatment of AGR mice grafted with non-lesional skin from psoriasis patients with 1 mg mAb to human CD8 resulted in complete blockade of psoriasis development, on par with the effect observed with TNF-α inhibitors^40^. These results suggest that CD8+ T cells may be a potential drug target for reducing TNF-α/IL-17A mediated inflammation.

In our differential expression analysis between the IL17A_Hi_ and the IL17A_Lo_ producing CD8+ T cells, we found that *TMPRSS11D* and *PM20D1* were down-regulated in the IL17A_Hi_, which Suarez-Farinas *et al*.^41^ observed to be down-regulated in lesional psoriasis skin versus healthy control skin. One of the most down-regulated genes in IL17A_Hi_ was *IL36B*, a cytokine that Li *et al*.^12^ discovered down-regulated signature keratinocyte genes. Interestingly, we did not find evidence of differential expression of *IL17A*, *IL13*, *IFNG*, or *IL22* (*TNF* was not detected in our RNA-seq analysis). We believe that bulk RNA-seq does not provide sufficient resolution to detect cytokine expression differences across different T cell populations. Indeed, we have recently shown^42^ that populations such as CD8+ T cells are not well represented in bulk RNA-seq analysis of skin tissue.

### T cell production of IL-22

IL-22 is an inflammatory cytokine produced Th22 cells that facilitates the recruitment of immune cells to the skin and disrupts keratinocyte maturation, causing acanthosis and parakeratosis in psoriatic skin lesions^31^. Although TNF-α promotes the maturation of Th22 cells and previous studies have found elevated IL-22 in psoriasis lesions, scalp psoriasis was the only subtype with significantly higher IL-22 and this difference was only significant in the IL-22 derived from CD8+ T cells. This suggests that IL-22 may have different roles in the inflammatory milieu depending on the plaque subtype, which may be due to site-specific variations in the cutaneous microflora and immunobiology^43^. For example, hair follicle cytokines are thought to modulate memory T cell homeostasis^44^. In addition, immune microenvironments are present along the follicle and peri-follicular tissue^45^. This contrasts with findings from a transcriptomic study of lesional and non-lesion psoriatic skin which found similar levels of IFN-γ expression in scalp and non-scalp lesions^14^. Non-scalp lesions also had a significantly higher activation of genes involved in the TNFα/IL-17/IL-22-induced keratinocyte responses, although the production of IL-22 from CD8+ T cells was not quantified. Furthermore, this study sampled scalp and non-scalp lesions from the same subject while our study took scalp and non-scalp lesions from different patients^14^. Alternatively, systemic anti-TNF-α treatment, which is known to reduce IL-22^31^, may be suppressing IL-22 levels in some patients.

### T cell production of IL-13

The decrease in CD4+ T effector-derived IL-13 seen in all subtypes of psoriasis is consistent with previous studies and suggests a shift in the Th1-Th2 axis. IL-13 is a cytokine produced predominantly by the Th2 lineage of CD4+ T cells^27^ and IL-13 polymorphisms are associated with altered psoriasis susceptibility^28^. In keratinocytes, IL-13 functions as a negative regulator of Th1 associated IFN-γ and TNF-α pathways via activation of STAT6, SOCS1, and SOCS3^30,31^. IL-13 has also been shown to inhibit the development of the Th17 subset of effector CD4+ T cells, which are thought to be a major source of IL-17 in psoriatic inflammation^32,33^. A reduction in IL-13 dependent suppression of Th1 cytokines and a concurrent increase in Th17 cells may help drive psoriatic inflammation^27^. The shift towards a Th1 dominated cytokine profile is supported by elevated TNF-α and IL-17A production from CD4+ T effector cells in psoriasis samples.

### T cell production of IFN-γ

Conventional plaque psoriasis was the only subtype to demonstrate significantly higher levels of IFN-γ compared to control skin. Elevated CD4+ T effector derived-IFN-γ is consistent with an immune response dominated by Th1 cells, which are the predominant producers of IFN-γ. However, CD8+ T cells also appear to produce higher levels of IFN-γ in psoriatic skin. The source of CD8+ T cell-derived IFN-γ may be the IL-17 secreting CD8+ T cells, which have demonstrated a similar capacity to produce IFN-γ^29,30^. This shift of CD8+ T cells towards a Th1 cytokine profile may represent an important pathologic mechanism in the development of psoriasis.

### Study limitations

One of the limitations for our study was the comparison of psoriasis subtypes to healthy control skin from the trunk and extremities. While this did have the advantage of allowing for a comparison of psoriasis subtypes to a common, uniform baseline, it also allows for the possibility that some DEG were detected on the basis of site-specific differences. While we were able to compare our list of DEGs from SP vs C against the the list of up-regulated genes in SP (relative to normal scalp tissue) from Ruano *et al*. and determined that excluding KRT- or KRTAP- genes may be appropriate as none of these genes were up-regulated in SP in Ruano *et al*.^14^, this comparison did not include genes that were down-regulated in SP (relative to normal scalp tissue). Finally, due to our limited sample size and sequencing batch effects, we were not able to stratify our analyses by sex. Future studies with sufficient numbers of gender-balanced samples within each subtype of psoriasis are needed to bolster the findings of this present study.

## Conclusion

The results of our study demonstrate that conventional, scalp, and palmoplantar psoriasis share a core set of molecular features, but also exhibit novel differences in gene expression, gene networks, T cell populations, and T cell cytokines. Understanding differences in the immunobiology between clinical subtypes can help us provide more effective, personalized treatment for psoriasis patients.

## Electronic supplementary material

Supplementary Figures

Supplementary Table 1

Supplementary Table 2

Supplementary Table 3

Supplementary Table 4

Supplementary Table 5

Supplementary Table 6
