# Supplementary material for: RNA-seq and flow-cytometry of conventional, scalp, and palmoplantar psoriasis reveal shared and distinct molecular pathways
Source: Sci Rep. 2018 Jul 27;8:11368. doi: 10.1038/s41598-018-29472-w (PMC6063960; doi:10.1038/s41598-018-29472-w)

RNA-seq and flow-cytometry of conventional, scalp, and palmoplantar psoriasis reveal shared and distinct molecular pathways

Richard Ahn[1*+], Di Yan[1,2*], Hsin-Wen Chang[1], Kristina Lee[1], Shrishti Bhattarai[1], Zhi-Ming Huang[1], Mio Nakamura[1], Rasnik Singh[1], Ladan Afifi[1], Keyon Taravati[1], Priscila Munoz-Sandoval[1], Mariela Pauli[1], Michael D. Rosenblum[1], Wilson Liao[1]

[1]Department of Dermatology, University of California, San Francisco, San Francisco, CA, United States.

[2]School of Medicine, Case Western Reserve University, Cleveland, OH, United States.

[*]Authors contributed equally to this paper

[+]Correspondence should be addressed to Richard Ahn, PhD, Department of Dermatology, University of California, San Francisco, 2340 Sutter Street, Box 0808, San Francisco, CA 94143-0808, USA (email: richard.ahn@ucsf.edu)

Supplementary Figure 1

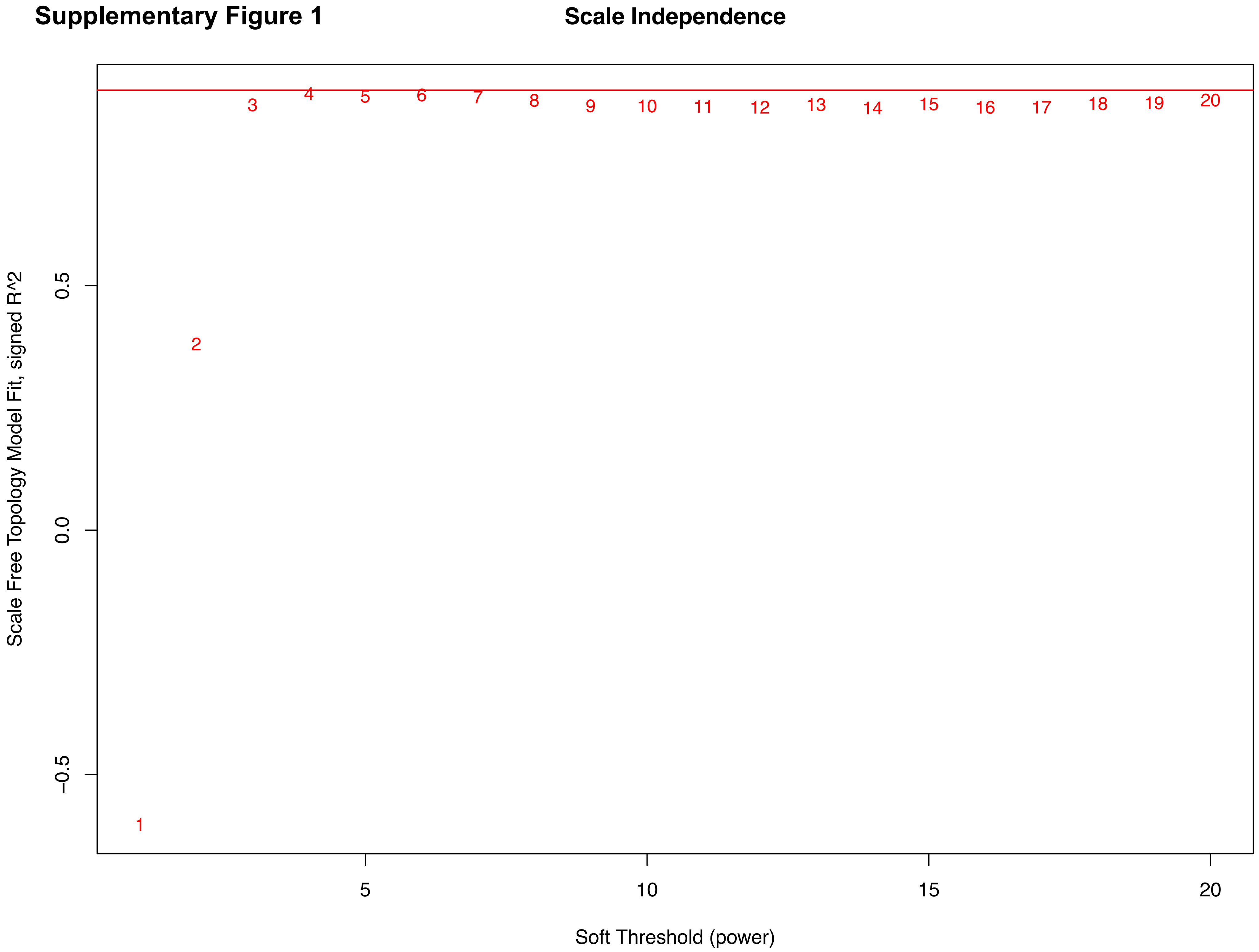

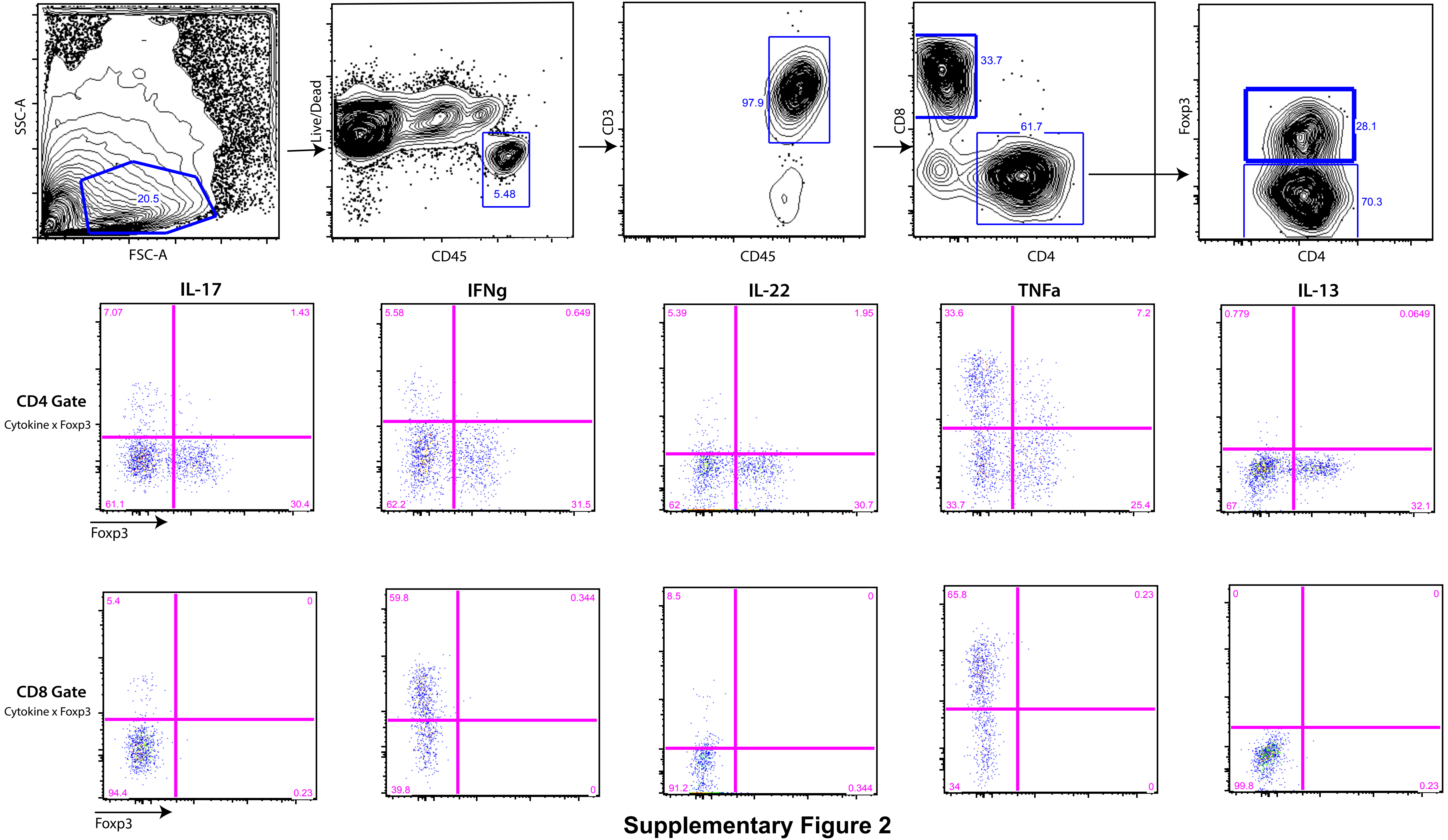

Supplementary Figure 2

Supplementary Figure 3 **Volcano plot of IL17A High vs Low in CD8s**

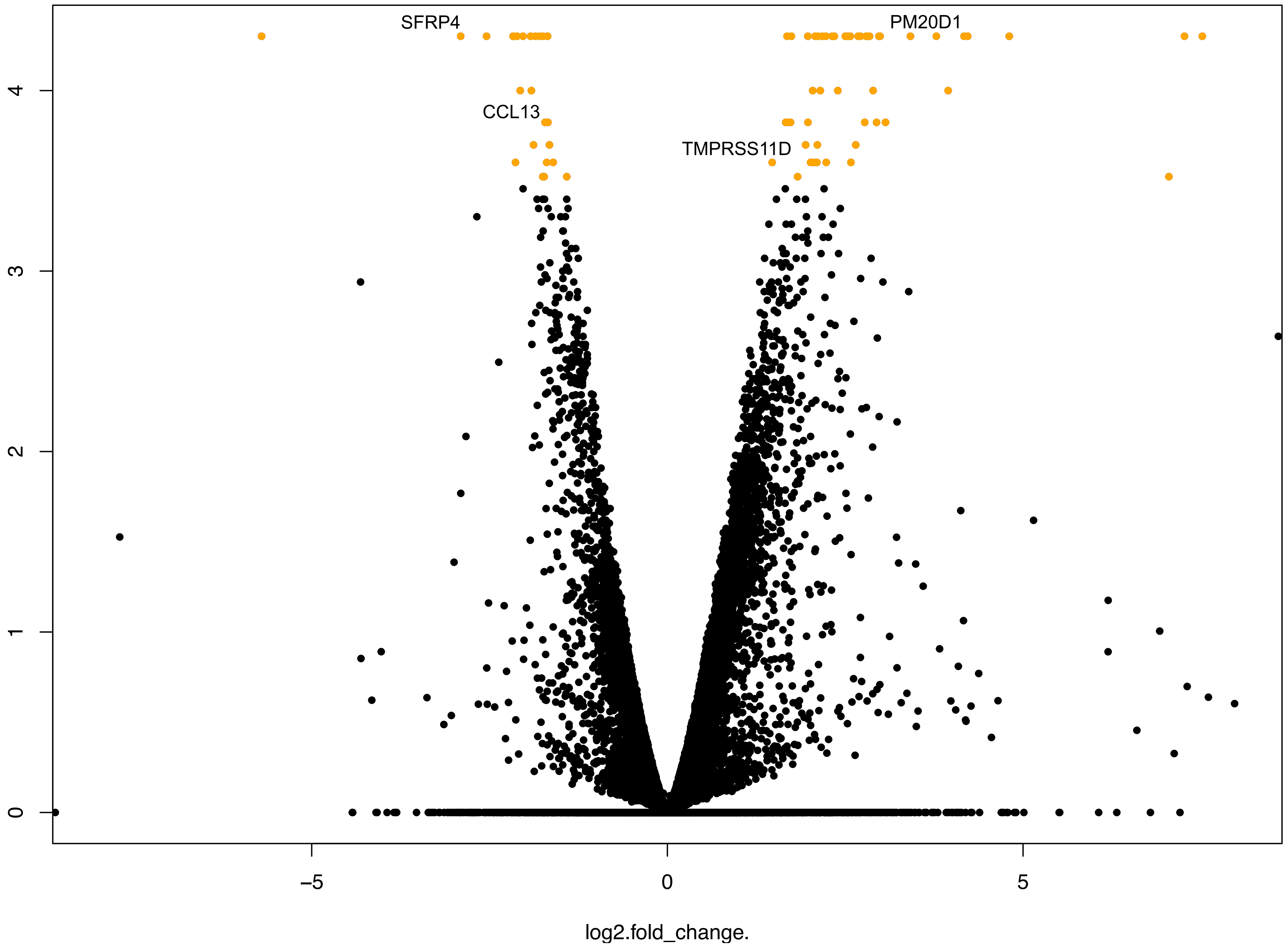

Supplement: Supplementary file 1 — Supplementary Figures [file 41598_2018_29472_MOESM1_ESM.pdf]
